# Characterization of a *nifH*-Harboring Bacterial Community in the Soil-Limited Gotjawal Forest

**DOI:** 10.3389/fmicb.2019.01858

**Published:** 2019-08-13

**Authors:** Tae Kwon Lee, Il Han, Min Sung Kim, Hoon Je Seong, Jong-Shik Kim, Woo Jun Sul

**Affiliations:** ^1^Department of Environmental Engineering, Yonsei University, Wonju, South Korea; ^2^Department of Systems Biotechnology, Chung-Ang University, Anseong, South Korea; ^3^Marine Industry Research Institute for East Sea Rim, Uljin, South Korea

**Keywords:** nitrogenase, *nifH*-harboring bacterial communities, cation-exchange capacity, co-occurrence network, Gotjawal forest, basalt rocks

## Abstract

Using a high-throughput metagenomic approach, we evaluated *nifH*-harboring bacterial communities and their assembly in the Gotjawal forest, which was naturally formed on basalt rocks with thin layer of soil. Significant differences in soil properties and community structure were observed in comparison with similar communities in various habitats, including other lava-formed forests (on Jeju Island and in Hawaii) and in regions with high humidity (Florida) or low temperatures (Alaska). *nifH*-harboring bacterial communities were found to assemble along gradients of environmental factors, particularly cation-exchange capacity. Unlike in other regions, in the Gotjawal forest, *Paenibacillus* and *Clostridium*, which belong to the phylum *Firmicutes*, were present in significantly higher proportion than in other regions. Network analysis suggested that much fewer co-occurrence relationships occurred in the Gotjawal forest than in other lava-formed forests. Our results indicate that the unique *nifH*-harboring bacterial community and its assembly in the Gotjawal forest are due to its distinctive soil properties, which has implications for microbial interactions and functional potentials.

## Introduction

The Gotjawal is a unique natural forest ecosystem that covers approximately 12% (224 km^2^) of Jeju Island, Korea. Owing to unregulated construction and urbanization, 50% of the Gotjawal forest has been destroyed in recent decades. The name “Gotjawal” is a complex term that describes the characteristics of these forests, which grow on basalt rocks with no soil or thin layer of soil; “Got” means “forest of thorny bush and trees,” whereas “Jawal” means “shallow soil wasteland” (Kang et al., 2013). The forest floor is made up of volcanic rocks and has hydrological properties essential for the ecosystem as it ensures rainwater percolation, thereby preventing water loss and maintaining constant temperature and high humidity throughout the year. These unique features create ideal conditions for the propagation of ferns and mosses. Trees rooted to the floor surface and fractured rocks on barren land rely on plants in the forest for nutrients. Mature trees in turn provide with a canopy, favoring the growth of the limited number of specialized plants found in the Gotjawal (Kang et al., 2013).

Trees in the Gotjawal forest are generally healthy and show no signs of nutritional deficiencies, indicating that they may be able to obtain nutrients solely from dry soil deposits. Since the development of the soil is poor and the surface and deep layer are mostly made up of large or small basalt rocks, it is difficult to settle easily even for the pioneer vegetation. A long period of time is necessary for the development of a forest such as found in the Gotjawal. Under soil-limited conditions, nitrogen fixation rates are affected by a pool of dissolved inorganic nitrogen, human activity, and local climatic conditions (Turk et al., 2011; Dias et al., 2012; Batterman et al., 2013; Espenberg et al., 2018). In this context, nitrogen fixation, the process via which plants regulate both nitrogen transformation and loss to the atmosphere, is particularly crucial for soil fertility. In addition, carbon and nutrient sources for microbial propagation and water for microbial dispersion are more restricted under soil-limited conditions, such as in deserts. These properties are postulated to decrease connectivity among habitats, thereby boosting separation of niches by reducing microbial interactions (Dechesne et al., 2010; Št’ovíèek et al., 2017).

Microorganisms play an indispensable role in maintaining a continuous supply of nitrogen for plants via nitrogen fixation (Mus et al., 2016). The diversity of nitrogen-fixing genes and diazotroph populations can be assessed by sequencing *nifH*, which encodes one of the subunits of the nitrogenase complex (Mus et al., 2016). Sequencing of *nifH* of various microbes has revealed sufficient variations in studies of nitrogen-fixing bacterial communities in various ecosystems as well as the measurement of alterations in their structure, stimulated by different soil properties and plants. However, the patterns and dynamics of *nifH*-harboring microbial communities under soil-limited conditions are yet to be systematically elucidated. In addition, the impact of environmental factors on *nifH*-harboring communities has not been evaluated based on the meta-community theory and co-occurrence microbial networks, which may help clarify and quantify the degree of species variation or biotic assemblage (Leibold and Mikkelson, 2002; Röttjers and Faust, 2018).

The meta-community theory aims to understand variations or dispersal in a community based on environmental variables (Leibold and Mikkelson, 2002). This theory can be used to quantify the degree of variation in a community and elucidate community patterns by linking species interactions to independent environmental gradients. Previous studies performed using the meta-community theory have reported that gradients of environmental factors, such as dryness, dissolved inorganic matter, and temperature, highly influenced bacterial community assembly in salt sediments, artic lakes, and seawater, respectively (Adams et al., 2014; Valverde et al., 2014; Ren et al., 2019). Community patterns can be illustrated by the three elements of the meta-community structure (i.e., coherence, turnover, and boundary clumping), and that information can then be used to delineate meta-community types (Leibold and Mikkelson, 2002). Six meta-community types (i.e., random, checkerboard, nested, evenly spaced, Gleasonian, and Clementsian) can be identified based on the community variance patterns. These types suggest that species responded either to environmental variation or to other parameters. Recently, the understanding of community assembly based on the meta-community theory has been reported to be critical for inferring the functions of bacterial communities (Griffin et al., 2017). Despite the reported importance of this approach, the meta-community theory has very rarely been applied in the context of a functional bacterial community.

The present study investigated novel features of *nifH*-harboring bacterial communities of the Gotjawal forest. The soil-limited conditions and unique physicochemical properties of this forest may affect the interactions of microorganisms with plants and microbe, thereby creating a distinct *nifH*-harboring community. The key objectives of the present study were as follows: (i) to compare soil physicochemical properties and *nifH*-harboring communities between the Gotjawal and forests of other regions with different soil characteristics and climate conditions, (ii) to evalute if the assembly of *nifH*-harboring communities are influenced directly by the soil physicochemical properties through the meta-community analysis, and iii) to determine whether the unique soil properties of the Gotjawal affect not only the abundance of *nifH*-harboring communities but also the microbial interactions within the community by bacterial co-occurrence network analysis.

## Materials and Methods

### Study Site and Soil Sampling

**FIGURE 1:**
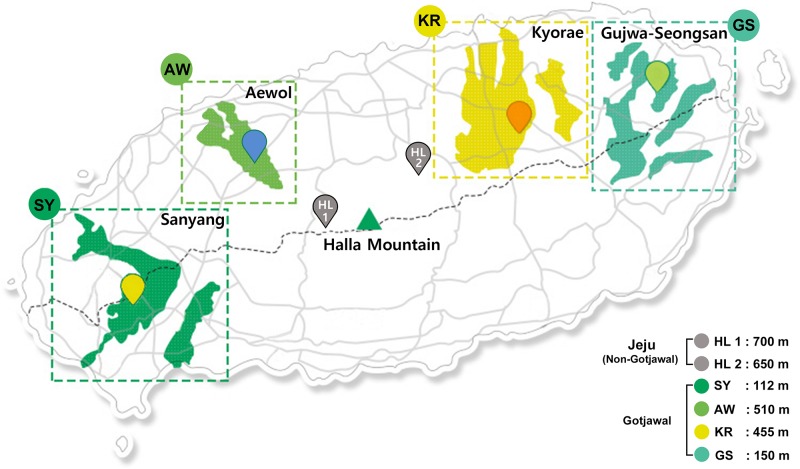
Location of Gotjawal (AW, KR, GS, and SY) and non-Gotjawal (HL1 and HL2) areas in Jeju Island, South Korea. The areas painted in greenish colors indicate the distribution of each Gotjawal area. The points represent the sampling locations.

The Gotjawal forest is located in a nature reserve on Jeju Island, Korea. The annual average temperature and humidity in the forest are 15.5°C (approximately 12.4°C–18.7°C) and 73% (approximately 70%–75%), respectively (Korea Meteorological Association). Four sampling sites were chosen to represent the Gotjawal forest: Sanyang (SY), Aewol (AW), Kyorae (KR), and Gujwa-Seongsan (GS). Also, two non-Gotjawal forests on the island (HL1 and HL2), which have a thicker soil layer, were selected for comparison (Figure 1). There is little soil or the depth of soil is relatively shallow in the Gotjawal. Soils were collected at places where the soil layer was formed over a certain depth and where the vegetation (e.g., mainly covered with diverse epiphytes and pteridophytes) was not mixed. Six composite soil samples were collected from within a 10 × 10 m area at each site (Figure 1) for four consecutive seasons. Samples were collected in the spring, summer, and winter (2016). Two areas (SY and KR) were additionally sampled in the fall of 2016. The soil samples were transported in an ice box to the laboratory and stored at −80°C for DNA extraction. Soil physicochemical properties (11 properties; pH, OM, K, Ca^+^, Mg^2+^, Na^+^, CEC, BCS, TOC, TN, and CN) were analyzed according to standard methods (National Academy of Agriculural Science [NAAS], 2010). Soil pH was analyzed using a pH meter (Orion Star A211, Thermo Fisher Scientific, United States) in a suspension of 5 g soil in 25 mL distilled water. Organic matter (OM) was measured using the Walkley–Black method (Walkley, 1935). Total organic carbon (TC) was indirectly calculated using the conversion factor (1.724). Total nitrogen (TN) was determined by the Kjeldahl method (Kjeltec analyzer unit, Foss, United States) (Bremner, 1965) after digestion of the soil with sulfuric acid. A carbon to nitrogen ratio (CN) was calculated using a ratio of the TC to TN. Exchangeable cation contents and cation-exchange capacity (CEC) were measured in an ammonium acetate solution (pH 7.0) using ICP-OES (JY 138 Ultrace, Jobin Yvon, United States) and the Chapman method (Chapman, 1965), respectively. The base cation saturation (BCS) ratio was calculated as a percentage of the CEC by dividing the sum of the base cation concentration by the CEC and multiplying it by 100. All soil physicochemical properties summarized in Supplementary Table S1 and detailed in Supplementary Table S2.

### DNA Extraction, Amplification, and Sequencing

Total genomic DNA was extracted using 0.25 g of each sample soil with the Fast DNA SPIN Kit for Soil (QBiogene Inc., Vista, CA, United States). The primer pair PolF/PolR (Poly et al., 2001), which amplifies *nifH*, was used for PCR amplification with an Illumina overhang adapter. All amplifications were performed in 25 μl total volume using the following mixture: 5 μl of Amplicon PCR forward and reverse Primer (5 pM), 12.5 μl of 2 × KAPA HiFi HotStart ReadyMix, 5 μl of PCR grade water and 2.5 μl of DNA (10 ng). The PCR cycling program for bacterial *nifH* was as follows: 95°C for 3 min; 25 cycles of 95°C for 30 s; 55°C for 30 s; 72°C for 30 s; 72°C for 5 min. The index PCR was carried out under the same conditions as above except that 8 cycles of amplification were used. The purified PCR products were quantified according to the qPCR Quantification Protocol Guide (KAPA Library Quantity Kit for Illumina Sequencing Platform) and verified using the LabChip GX HT DNA High Sensitivity Kit. Sequencing was performed with paired-end sequences (2 × 300 bp) with Macrogen quipped with the MiSeq^TM^ platform (Illumina, San Diego, United States).

### Sequence Processing

MiSeq paired reads of *nifH* amplicons from the Gotjawal, non-Gotjawal, and NEON samples (see point “Data Collection and Description of Sample Characteristics” below) were assembled using the RDP Initial Process tool (Cole et al., 2013) with a minimum overlap of 10 bp and a length filter between 300 and 400 bp. Six samples of Gotjawal, which produced less than 500 reads, were excluded from the analysis. Assembled reads were filtered using SeqFilter for reads without N numbers with a minimum quality score of 20 and forward/reverse maximum primer mismatches equal to 1. Selected reads were classified with RDP FrameBot (Wang et al., 2013) to correct frameshift errors and assigned to the closest match from a curated *nifH* set (Wang et al., 2013) at a cutoff of 50% amino acid sequence identity. The number of reads obtained throughout each filtering processes were summarized in Supplementary Table S3. Protein reads were aligned and clustered using complete linkage clustering methods at 5% amino acid dissimilarity. The representative sequences were selected using the minimum sum of the square of the distance within each cluster. The curated *nifH* set was used as the reference set for BLASTp analyses. The OTU richness, Shannon, and Evenness were used for *nifH* diversity estimates.

### Co-occurrence Network Construction

Using *nifH*-harboring bacterial community data, we analyzed microbial networks for each soil sample from the Gotjawal forest and two different sets of lava-formed forests (HL1, HL2, and HI). Co-occurrence networks were inferred based on Spearman correlation matrix and constructed using only significant correlations (Barberán et al., 2012; Ma et al., 2016). The cutoff for correlation coefficients (*R*-value) was 0.8, whereas that for *P*-values was 0.001. Cutoff values were selected based on variance of interaction strength. All network construction steps were calculated using the R program with a code adapted from https://github.com/ryanjw/co-occurrence. We generated network images of three communities with the same node arrangement by fixing layout, calculating community density using the edge density function, and analyzing clustering coefficient using the transitivity function. Visualization of microbial community networks and calculation of network properties were performed using the R package igraph software (Csardi and Nepusz, 2006).

### Meta-Community Structure Analysis

The R package metacom package was used to assess the elements of meta-community structure frameworks that enable differentiation of meta-communities into nine best-fit idealized structures by examining patterns in the distribution of 185 genera in three stages: coherence, species turnover, and range boundary clumping (Presley et al., 2010; Dallas, 2014). R0 methods were used to run 1,000 null models for comparison against our observed matrix (using a presence–absence species-by-site matrix). Site scores were then calculated based on the primary RA axis as a representative of the meta-community organization within each treatment and correlated with soil physicochemical properties using Spearman correlation (Dallas, 2014).

### Statistical Analyses

All statistical analyses were performed using the R package vegan software (Oksanen et al., 2013). Differences in multivariate aspects of *nifH*-harboring bacterial communities and soil properties were determined via non-metric multidimensional scaling (NMDS) using the Bray–Curtis similarity index. Soil properties were fitted as vectors using the R package vegan software. Among 11 soil properties, seven properties (CEC, Na^+^, Mg^2+^, Ca^2+^, BCS, CN, and K^+^) were selected for the major loading vectors contributing the ordinations by permutation test (*p* < 0.05). The significance of the differences was evaluated by comparing *t*-test or Wilcoxon ranked sum test according to the normality test by Shapiro-Wilks test. Analysis of similarity (ANOSIM) was performed to assess whether the soil properties or community structures were significantly different across ecosystems. Correlations between the community structure and soil properties were tested using Spearman correlation.

### Data Collection and Description of Sample Characteristics

Data of 216 *nifH* amplicon sequences were obtained from National Ecological Observatory Network (NEON^1^) sites (Alaska, AK; Florida, FL; and Hawaii, HI) from the ENA Short Read Archive under the accession numbers ERP002231, ERP002028, ERP002042, and ERP002032; soil metadata were obtained from the Supplemental Material of Wang et al. (2013). NEON *nifH* amplified sequence data were analyzed as described above. The ecological regions of each site were boreal (poor drainage), subtropical/dry (excessive drainage), subtropical/lower montane wet forest (moderate drainage) for AK, FL and HI, respectively. We selected 3 of the NEON observatories representing very different soil characteristics and climate conditions. The detailed characteristics of each site are well documented in the previous reports (Wang et al., 2013).

### Nucleotide Sequence Accession Numbers

The sequences reported in this study were deposited in the NCBI Sequence Read Archive under the accession numbers PRJNA516305.

## Results

### Soil Physicochemical Properties

**FIGURE 2:**
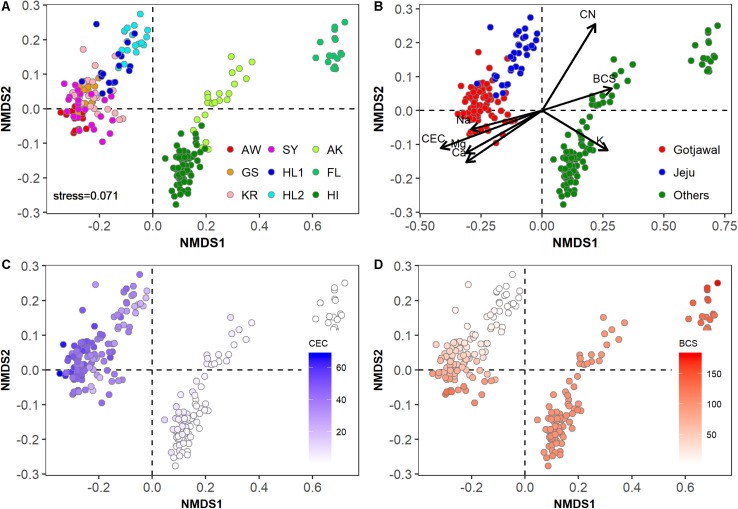
Non-metric multidimensional scaling (NMDS) plot showing physicochemical properties in soil samples in this study (*k* = 2; stress = 0.0741). **(A)** Individual sampling points, **(B)** four regions (AW, KR, GS, and SY) of Gotjawal: Gotjawal, Non-Gotjawal forest on Jeju Island (Jeju), and other regions (Others), **(C,D)** concentrations of CEC and BCS are indicated by the color gradient from white to blue and red, respectively. The soil properties indicated by arrows in **(B)** are the significant properties (*p* < 0.05) contributing to each ordination.

The physicochemical properties of the Gotjawal forest soil samples (SY, AW, KR, and GS) were significantly different from those of the non-Gotjawal forest soil samples from Jeju Island (HL1 and HL2) and from those of regions outside of South Korea (AK, FL, and HI) (Figures 2A,B). The Gotjawal forests tightly clustered regardless of regions and seasons (Figure 2A). The soil properties including cations (Ca^2+^, Mg^2+^, and Na^+^), CEC and BCS were significantly different from non-Gotjawal forest and other regions (*p* < 0.05; Supplementary Table S1). These properties were also selected as significant loading vectors (*p* < 0.05) contributing the ordination in the NMDS plots (Figure 2B). In particular, there were regional differences (all three regions were statistically different; *p* < 0.05) in the CEC and BCS (Supplementary Table S1 and Figures 2C,D). The CEC level on Jeju Island, including both the Gotjawal and non-Gotjawal forests, was significantly higher (*p* < 0.01) than in other regions. The BCS was highly variable among soil samples; most notably, it was significantly lower in non-Gotjawal forests on Jeju Island than in other regions (*p* < 0.01). These results reveal that the Gotjawal forest has a unique ecosystem with exclusive soil physicochemical properties, particularly in terms of CEC and BCS.

### *nifH*-Harboring Community Structure and Phylogenetic Composition

**FIGURE 3:**
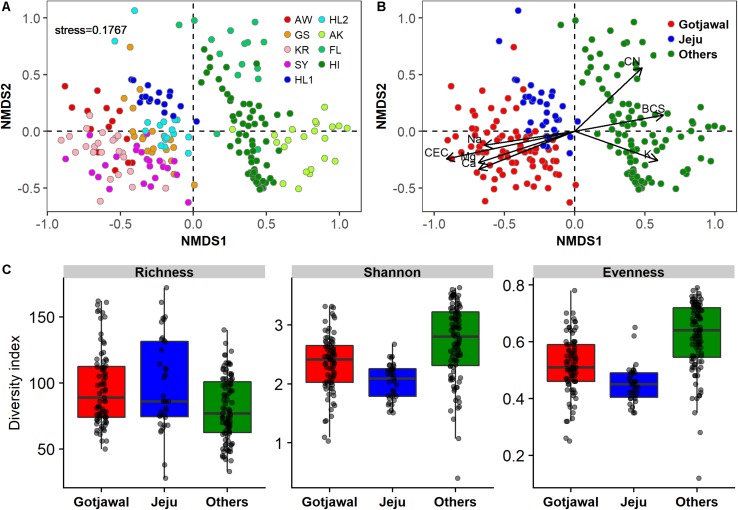
Non-metric multidimensional scaling (NMDS) plot showing the *nifH*-harboring communities present in this study (*k* = 2; stress = 0.1767). **(A)** Individual sampling points, **(B)** four regions (AW, KR, GS, and SY) of Gotjawal: Gotjawal, Non-Gotjawal forest on Jeju Island (Jeju), and other regions (Others). **(C)** Microbial diversity of three regions. The soil properties indicated by arrows in **(B)** are the significant properties (*p* < 0.05) contributing to each ordination.

There were no seasonal or spatial differences in the *nifH*-harboring bacterial communities within the Gotjawal, but the structure of the Gotjawal communities differed from that of communities in the non-Gotjawal forest on Jeju Island and in other regions according to NMDS ordinations (Figures 3A,B). ANOSIM analysis revealed an *R*-value of 0.82 and a *P*-value of < 0.001, and these values were supported by shifts observed in the values of seven physicochemical properties (CEC, Na^+^, Mg^2+^, Ca^2+^, BCS, CN, and K^+^; *p* < 0.01 for all). Along with CEC, Na^+^, Mg^2+^, and Ca^2+^ were positive and significantly associated with the *nifH*-harboring bacterial communities of the Gotjawal forest, whereas CN, BCS, and K^+^ were associated with the communities in the other regions studied. Although the community structures differed, the alpha diversities of *nifH* were not found to be significantly different among sites (Figure 3C).

**FIGURE 4:**
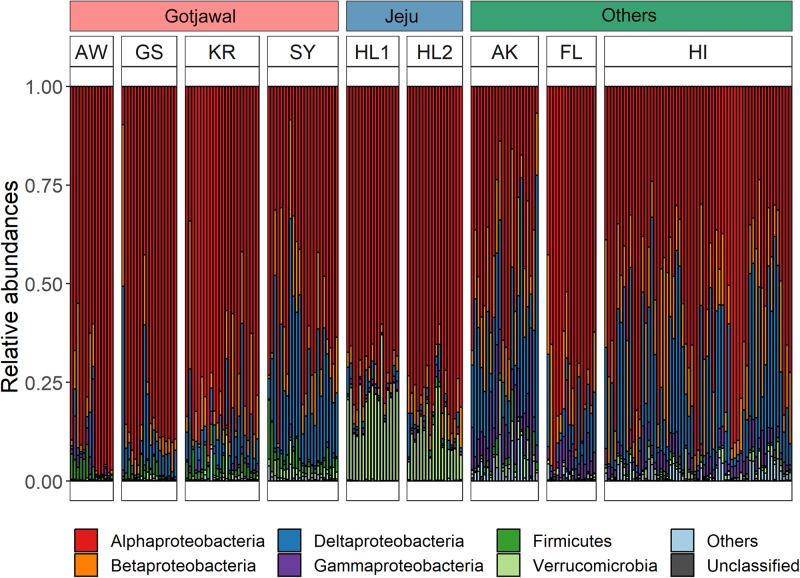
Bar graphs indicate six dominant phyla in three regions. Each bar of Gotjawal and Jeju are ordered by seasons. Others are Alaska (AK), Florida (FL), and Hawaii (HI) regions, where the soil layer is relatively thick, and represent boreal, subtropical/dry, and lower montane wet forest, respectively. Each phylum is indicated in a different color.

Overall, 15 different *nifH*-harboring bacterial communities were identified from the soil samples. Among these, six bacterial phyla (Alpha-, Beta-, Delta-, and Gammaproteobacteria; Firmicutes; and Verrucomicrobia) were predominant (88.2%–100%; Figure 4). Statistical analysis (*t*-test) confirmed significant differences (*p* < 0.05 for all) in these phyla between the Gotjawal forest and other regions; however, high similarity was observed between the Gotjawal and the non-Gotjawal forests of Jeju Island (except for Deltaproteobacteria, Firmicutes, and Verrucomicrobia). The primary difference was observed for Firmicutes, which were considerably more prevalent in the Gotjawal forest than in the other regions. Of note, only the SY sampling site showed a significantly different composition of nifH-harboring Deltaproteobacteria, Alphaproteobacteria, and Verrucomicrobia (Figure 4) compared to the other sites. Firmicutes, Betaproteobacteria, and Gammaproteobacteria were not significantly different among the Gotjawal forest samples.

In order to analyze the nitrogen fixing bacteria in Gotjawal more specifically, the genera with average relative abundance 0.5% or higher were selected for further analysis (Supplementary Figure S1). A total of 12 genera were selected and belong to Alphaproteobacteria (six genera; *Azospirillum*, *Bradyrhizobium*, *Gluconacetobacter*, *Hyphomicrobium*, *Xanthobacter* and *Rhizobium*), Betaproteobacteria (three genera; *Burkholderia*, *Dechloromonas*, and *Rubrivivax*), Deltaproteobacteria (one genus; *Geobacter*) and Firmicutes (two genera; *Clostridium* and *Paenibacillus*). The relative abundances of these members were highly predominant regardless of the regions (The Gotjawal: 72.6−99.1%, Jeju: 60.6−90.5%, and Others: 23.9−97.9%). Among those members, four genera including *Clostridium*, *Dechloromonas*, *Gluconacetobacter*, and *Paenibacillus* in the Gotjawal had significantly higher relative abundance in the Gotjawal compared to non-Gotjawal and other regions (*p*-value < 0.05). As shown in the results at phylum level, the two genera belonging to Firmicutes seem to be uniquely distributed in Gotjawal regions unlike other regions.

### The *nifH*-Harboring Meta-Community Assembled With Soil Properties

**FIGURE 5:**
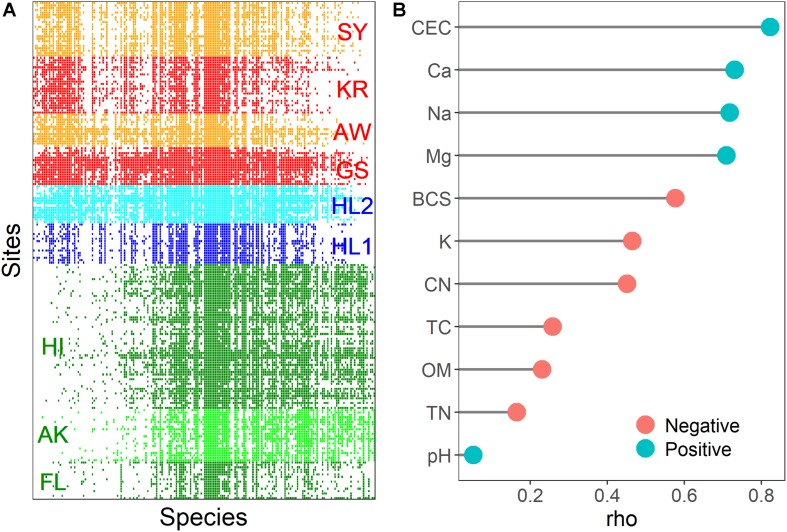
A visualization of the meta-community analysis with sites as rows and OTUs as columns **(A)**. The colored rectangles (red: Gotjawal, blue: Jeju, green: Others) indicate a OTUs occurrence at a site. Spearman coefficients (rho value) of the soil physicochemical properties associated with the structuring gradient species distribution **(B)**. The red and blue colors of the circle indicate negative and positive correlation between soil properties and site scores, respectively.

The meta-community of the *nifH-*harboring bacterial community contained fewer embedded absences (Coherence; *z* = 138.63, *p* < 0.001) and more species replacements than expected (Turnover; *z* = −1.13; *p* < 0.001), and species range boundaries were significantly different from the null expectation (Clumping; Moristita’s index = 6.12; *p* < 0.001). Based on these results, the meta-community would traditionally be considered Clementsian (community assembly along environmental gradients), i.e., the boundaries of species ranges are highly coincident. The community matrix used to visualize meta-community occurrence patterns is illustrated in Figure 5A. All sites shared the 18 *nifH* OTUs (9.3% of total *nifH* OTUs) assigned to Alphaproteobacteria (*Azospirillum*, *Rhizobium*, *Bradyrhizobium*, *Hyphomicrobium*, and *Xanthobacter*), Betaproteobacteria (*Burkholderia*, *Leptothrix*, and *Polaromonas*), Deltaproteobacteria (*Geobacter* and *Pelobacter*), and Verrucomicrobia (*Optitutaceae bacterium*). The total relative abundance of these phyla was associated with CEC and cations (rho = 0.306 and *p* < 0.05 in the Gotjawal forest; rho = −0.323 and *p* < 0.05 in other regions). There were 14 *nifH* OTUs (7.3% of total *nifH* OTUs) assigned to Firmicutes (*Clostridium* and *Paenibacillus*) observed only in the Gotjawal forest. No correlations were observed between the soil properties and the relative abundances of these Firmicutes *nifH* OTUs.

The site and species score, which is an interaction matrix score derived from reciprocal averaging (Gauch et al., 1977), can be assessed with soil physicochemical properties to provide evidence of the importance of these properties in structuring species distribution. We used non-parametric Spearman correlation to identify associations between the site scores obtained from reciprocal averaging and subsets of soil physicochemical properties. The result showed that most soil physicochemical properties were associated with the structuring of *nifH*-harboring communities, although pH was not correlated with site scores (Figure 5B). The CEC and cations are potentially more important in determining *nifH*-harboring community composition at the study sites.

### Comparison of Co-occurrence *nifH* OTUs Networks in Lava-Formed Forests

**FIGURE 6:**
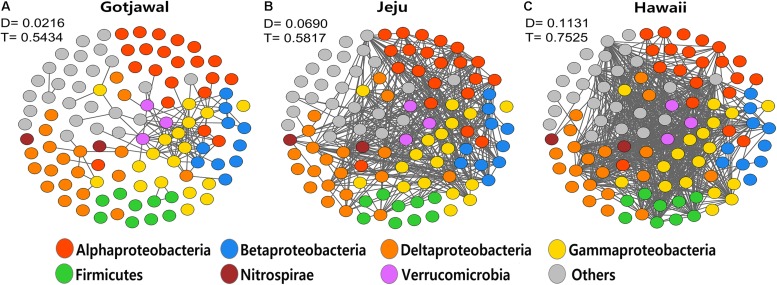
Network of co-occurring microbial genera based on correlation analysis for **(A)** Gotjawal, **(B)** forest on Jeju Island, and **(C)** Hawaii. A connection stands for a strong (Spearman *P* > 0.8) and significant (*P* < 0.01) correlation. Nodes are colored according to phylum. D, density; T, transitivity.

The co-occurrence networks of the *nifH-*harboring communities were significantly different between the observed lava-formed forests (Gotjawal and non-Gotjawal forests on Jeju Island and Hawaii). The transitivity (T) and the degree (D) of co-occurrence networks in the Gotjawal were noticeably lower than in other lava-formed forests (HL1, HL2, and HI). Furthermore, fragmentation of the networks was significantly higher in the Gotjawal than in other forests (Figure 6). A few genera belonging to the Gammaproteobacteria tended to coexist and play a role as hub nodes in the networks of the lava-formed forests. However, many nodes between genera did not appear and were mostly restricted in the Gotjawal (*D* = 0.0216; *T* = 0.5434), although the other lava-formed forests appeared to be very interconnected (*D* = 0.0690 and *T* = 0.5817 in HL1 and HL2; *D* = 0.1131 and *T* = 0.7525 in HI). This pattern was consistent for individual sites in the Gotjawal forest (Supplementary Figure S2).

## Discussion

Biological nitrogen fixation is a key ecosystem process influenced by resident functional microbial communities and environmental conditions. The Gotjawal forest may be a unique habitat with a nitrogen fixation process that is influenced by diazotrophs associated with a narrow range of plants (e.g., ferns and mosses) specialized to colonize such environments (mostly ferns, mosses, and trees). In the present study, we examined the diversity of *nifH* (encoding a subunit of the nitrogenase complex) in this unique forest as well as other lava-formed forests via statistical analyses and the meta-community theory. We determined that soil CEC and cations significantly contribute to the structuring of *nifH*-harboring bacterial communities. We also found that the fragmentation of the co-occurrence networks of *nifH*-harboring bacterial communities was significantly higher in the Gotjawal forest. Few previous studies have addressed the microbial diversity of the Gotjawal forest; however, those studies mostly focused on exploring taxonomical groups inhabiting this unique ecosystem and on the changes in these groups under different environmental pressures (Kim et al., 2018). Hence, the composition and dynamics of the functional groups in *nifH*-harboring bacterial communities has been limitedly explored and poorly understood.

*nifH*-harboring bacterial communities are widespread in the terrestrial environment. Their diversity and structures reportedly differ based on biogeographical features, such as soil physicochemical properties, plant types, fertilization rate, and soil management practices (Wang et al., 2013; Bouffaud et al., 2016; Fernández-Méndez et al., 2016). Both NMDS plots (Figures 2B, 3B) with the soil physicochemical properties and microbial communities showed that Gotjawal clustered differently compared to other regions and even non-Gotjawal areas in Jeju Island. Interestingly, the plot based on the soil physicochemical properties (stress: 0.0741) represented more regional characteristics than the plot based on the microbial communities (stress: 0.1767). These results indicate that the microbes that play a special role, such as nitrogen fixation, are coexistent regardless of spatial heterogeneity and their abundances were varied according to the soil properties. We also found that the *nifH*-harboring bacterial community responded to potential environmental gradients (particularly CEC and cations), thereby indicating positive species turnover but with individualistic responses. This finding is typical of a Clementsian structure, a type of meta-community shaped by habitat preferences (Tonkin et al., 2017). The Clementsian pattern suggests that *nifH*-harboring communities are determined via biological interactions, thereby reflecting biological symbiosis (Boucher, 1988). These results support the theory that limited and specialized plant types (e.g., epiphyte and pteridophyte) are the most important environmental factors influencing the structure of the *nifH*-harboring bacterial communities in the Gotjawal forest because nitrogen fixation is subtly regulated by nutritional status and symbiotic reactions with plants (Bouffaud et al., 2016). These effects would be similar depending on changes in vegetation in seasonal and soil characteristics in other regions, which have different types of vegetation (e.g., tundra biome for AK, tropical biome for HI, and complex biome for FL).

However, *nifH*-harboring communities showed less ordered variation (Figure 5) and individualistic OTU responses to multiple environmental gradients (Heino et al., 2015). Although we determined that biological gradients are the most important to the meta-community structure (Clementsian) and species along a latent gradient, the site score also strongly correlated with CEC, cations, BCS, TN, and OM. This could be explained by the strong selection of plant types based on soil physicochemical properties and climate (Espenberg et al., 2018). The surface of the volcanic rock with no soil or thin layer of soil in the Gotjawal forest only allows the growth of specific species of ferns and mosses, which are important sources of nutrients for tree roots on the rock surface or for those exposed in fractured rocks. As the forest matures, taller trees provide better shading and create an area of poor air circulation, constant temperature, and humidity for specialized plants to flourish (Kang et al., 2013).

Our results revealed a substantial overlap of dominant phyla in *nifH*-harboring bacterial communities between the sites compared in this study (Figure 4). Our findings implied a persistent and ubiquitous *nifH*-harboring bacterial community that was dominant regardless of the biotic and abiotic properties of the soil. Although the Gotjawal forest and the forests in other regions shared similar dominant phyla, they still showed significant differences in the *nifH*-harboring bacterial community structure. The present results demonstrate that soil physicochemical properties are generally dissimilar among sites, suggesting that the relative abundance of each phyla is influenced by soil physicochemical properties and plant types in each region (Wang et al., 2013; Bouffaud et al., 2016). All phyla (14 OTUs) correlated with the soil physicochemical properties, including CEC and cations. In contrast, the lack of correlation between Firmicutes *nifH* abundance (*Paenibacillus* and *Clostridium* at the genus level) and environmental gradients suggested a relative abundance of various plant types and a lack of microbial dispersal in a distinct area under soil-limited conditions (Št’ovíèek et al., 2017). The facultative anaerobic bacteria *Paenibacillus* and *Clostridium* were the main diazotrophs in areas with a variety of ferns and mosses. These genera were uniquely observed in our results in the Gotjawal (Supplementary Figure S1), and it was also consistent with previous results based on 16S rRNA gene amplicons suggesting that *Clostridium* and *Paenibacillus* were indicator taxa in the Gotjawal area (Kim et al., 2018). Therefore, we concluded that the relative abundance of dominant and ubiquitous members in *nifH*-harboring communities is strongly associated with soil physicochemical properties and that the bacteria observed exclusively in the Gotjawal forest are highly influenced by plant types because bacterial dispersion is restricted under soil-limited conditions.

Our results demonstrate that the Gotjawal network is less complex and coherent than the networks of other lava-formed forests. The Gotjawal network showed higher fragmentation of the *nifH*-harboring bacterial community than other regions. The higher taxonomic dissimilarity of *nifH*-harboring bacterial communities between the Gotjawal forest and other lava-formed forests probably explains why most of the nodes remained disconnected. These observations imply that a network is not a random combination of nodes but is rather organized as a habitat network with unique functional significance in a complex web of associations between plants and soil physicochemical properties. The poor quality of complex organic substrates in soil-limited forests may require microbial syntrophy from multiple species; further, the uptake and utilization of compounds would require more extracellular hydrolysis using microbial enzymes. However, such microbial syntrophy may be restricted by reduced microbial dispersion and nutrient transport under soil-limited conditions with low water-holding capacity (Makhalanyane et al., 2015). The ecological network theory also predicts that communities of tightly connected species in small niches should be more fragile (Montoya et al., 2006). Less connectivity under soil-limited conditions leads to robust niche partitioning and distinct bacterial communities generating new symbiotic relationships (Baran et al., 2015; Ho et al., 2017). Furthermore, flow-induced disturbance by intermittent rainfall could wash bacteria from the soil surface, thereby reducing symbiotic relationships and enhancing the tightness of persistent species in each niche (Allison and Martiny, 2008; Galand et al., 2016). Repeated disturbances could eliminate gatekeepers, which keep maintain network organization. Taken together, our findings suggest that the loss of gatekeepers due to niche partitioning and flow-induced disturbance in soil-limited disproportionately habitats to co-occurrence network fragmentation, which fundamentally agrees with previous reports on food web networks showing high fragility of the networks upon selective removal of species (Pocock et al., 2012). Analysis of *nifH*-harboring communities allowed us to identify gatekeepers (groups ubiquitous at all sites) within the Alphaproteobacteria, Betaproteobacteria, Deltaproteobacteria, and Verrucomicrobia. These members provided core nodes with high connectivity in the co-occurrence networks of the other lava-formed forests, but only the Gammaproteobacteria performed this role in the Gotjawal forest. These results also support the idea that gatekeepers are remarkably reduced by niche partitioning and disturbance in the Gotjawal forest, thereby contributing to adverse consequences for the integrity and function of *nifH*-harboring communities.

## Data Availability

The datasets generated for this study can be found in NCBI, PRJNA516305.

## Author Contributions

TL performed data analysis and wrote the manuscript. HS and MK performed laboratory work and processed sequencing data. WS, JK, and TL designed the study. WS and TL were involved in the organization and management of the overall project. WS, JK, and IH were involved in peer-editing of the manuscript.

## Conflict of Interest Statement

The authors declare that the research was conducted in the absence of any commercial or financial relationships that could be construed as a potential conflict of interest.

## Footnotes

1 www.neonscience.org
