# Supplementary material for: Characterization of a nifH-Harboring Bacterial Community in the Soil-Limited Gotjawal Forest
Source: Front Microbiol. 2019 Aug 13;10:1858. doi: 10.3389/fmicb.2019.01858 (PMC6700220; doi:10.3389/fmicb.2019.01858)
Supplement: Supplementary file 1 [file Data_Sheet_1.docx]

Supplementary Material

# Supplementary figures


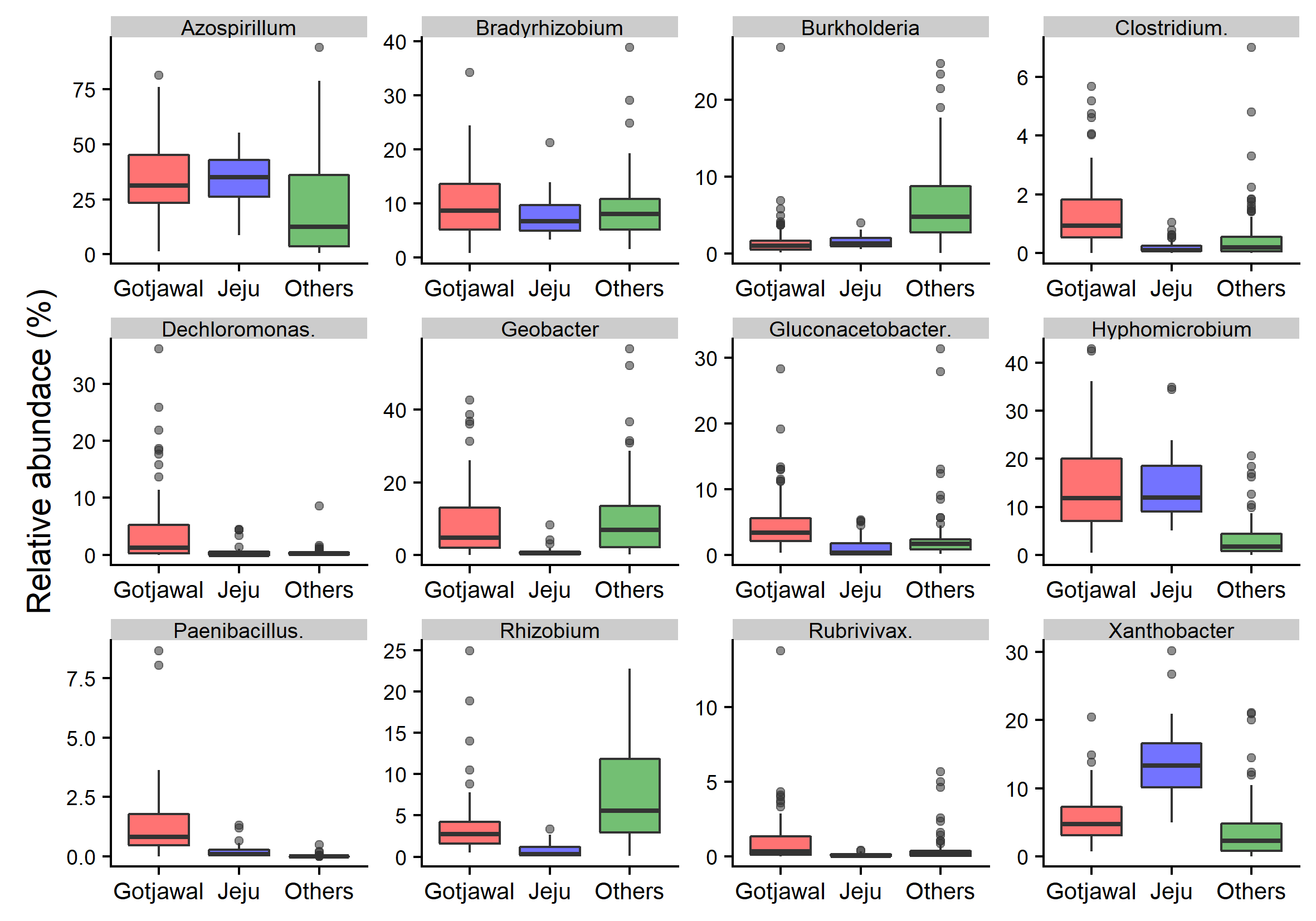


**Supplementary Figure 1.** Boxplots of bacterial genera with an average relative abundance of higher than 0.5% in the Gotjawal was indicated. *Clostridium*, *Dechloromonas*, *Gluconacetobacter* and *Paenibacillus* were significantly higher than non-Gotjawal and other regions (p < 0.05).

**
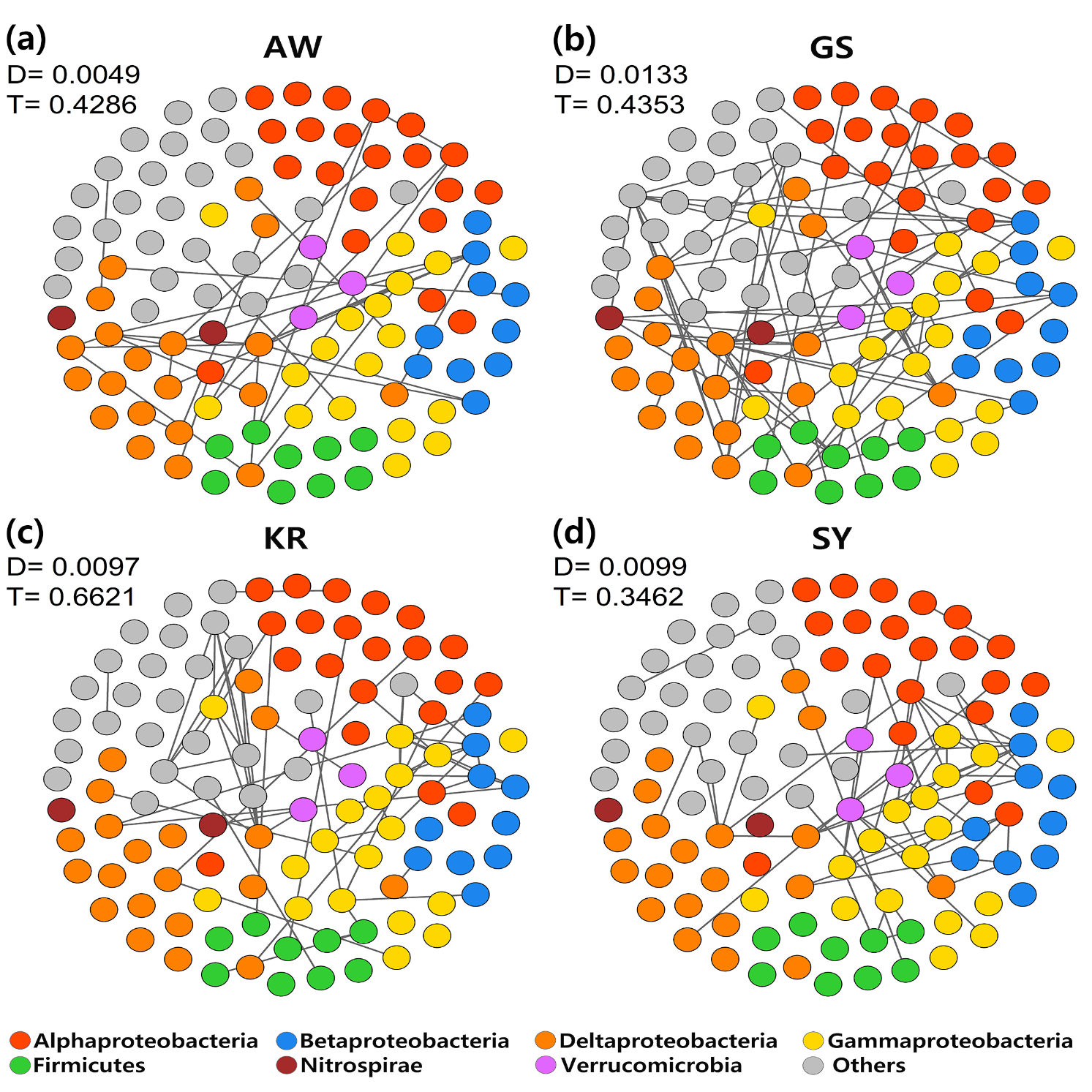
**

**Supplementary Figure 2.** Network of co-occurring microbial genera based on correlation analysis for **(A)** Gotjawal, **(B)** Forest on Jeju Island and **(C)** Hawaii. A connection stands for a strong (Spearman’s rho > 0.8) and significant (*P* < 0.01) correlation. Nodes are colored according to phylum. D: Density, T: Transitivity

Supplementary Tables

# Supplementary Table 1. Summary of soil physicochemical properties (mean± SD), averaged across all soil samples for each of the regions. Different letters in the same column indicate significant differences at p < 0.05.

| Region | pH | OM (%) | K (cmol/kg) | Ca (cmol/kg) | Mg (cmol/kg) | Na (cmol/kg) | CEC (cmol/kg) | BCS (%) | TC (%) | TN (%) | **CN** |
| --- | --- | --- | --- | --- | --- | --- | --- | --- | --- | --- | --- |
| Gotjawal | 5.0±0.5 a | 30.9±11.6 a | 0.8±0.3 a | 22.0±11.4 a | 4.6±2.6 a | 0.5±0.2 a | 45.4±9.4 a | 62.5±28.0 a | 17.9±6.8 a | 1.3±0.3 a | 13.5±2.7 a |
| Jeju | 4.4±0.3 b | 18.8±5.5 b | 0.4±0.2 a | 2.7±2.6 b | 1.0±1.0 b | 0.3±0.3 b | 34.9±8.2 b | 11.6±8.7 b | 10.9±3.2 b | 0.7±0.2 b | 15.4±3.7 a |
| Others | 4.8±0.7 a | 36.0±23.3 a | 2.7±2.0 b | 0.9±0.6 b | 0.2±0.1 b | 0.1±0.1 b | 3.6±2.5 c | 110.5±15.9 c | 22.7±15.1 a | 1.3±0.9 a | 20.9±7.0 b |

**Supplementary Table 2.** Soil physicochemical properties of Gotjawal and non-Gotjawal areas in Jeju Island. Sample names consist of sample abbreviations, seasons, and replicates. The roman numerals after the abbreviation of the sample mean the seasons sampled. I: Spring, II: Summer, III: Fall, IV: Winter. The number

|  | Sample | Code | ID | pH | OM  (%) | K  (cmol/kg) | Ca  (cmol/kg) | Mg  (cmol/kg) | Na  (cmol/kg) | CEC  (cmol/kg) | BCS  (%) | TC  (%) | TN  (%) | CN |
| --- | --- | --- | --- | --- | --- | --- | --- | --- | --- | --- | --- | --- | --- | --- |
| 1 | AWI_1 | Gotjawal | AW | 4.6 | 50.3 | 1.0 | 26.4 | 7.0 | 0.5 | 45.9 | 75.9 | 29.2 | 1.8 | 16.0 |
| 2 | AWI_3 | Gotjawal | AW | 4.5 | 49.1 | 1.0 | 25.6 | 7.4 | 0.4 | 52.9 | 65.2 | 28.5 | 1.5 | 18.4 |
| 3 | AWI_4 | Gotjawal | AW | 4.5 | 59.2 | 1.2 | 33.7 | 9.7 | 0.5 | 46.1 | 98.0 | 34.3 | 1.9 | 18.1 |
| 4 | AWI_5 | Gotjawal | AW | 4.9 | 56.7 | 1.3 | 31.5 | 7.6 | 0.5 | 41.3 | 99.1 | 32.9 | 1.8 | 18.7 |
| 5 | AWI_6 | Gotjawal | AW | 4.8 | 60.3 | 1.4 | 39.3 | 10.8 | 0.5 | 49.9 | 104.1 | 35.0 | 1.9 | 18.4 |
| 6 | AWII_1 | Gotjawal | AW | 4.7 | 42.8 | 0.9 | 25.2 | 6.5 | 0.5 | 53.1 | 62.4 | 24.8 | 1.3 | 18.9 |
| 7 | AWII_3 | Gotjawal | AW | 4.9 | 50.0 | 1.1 | 45.2 | 11.4 | 0.5 | 43.5 | 133.9 | 29.0 | 1.5 | 19.0 |
| 8 | AWII_5 | Gotjawal | AW | 4.7 | 34.8 | 0.8 | 48.9 | 6.3 | 0.4 | 43.1 | 130.8 | 20.2 | 1.3 | 15.9 |
| 9 | AWIV1 | Gotjawal | AW | 4.5 | 54.5 | 1.5 | 34.7 | 6.5 | 1.0 | 53.1 | 82.3 | 31.6 | 2.0 | 15.6 |
| 10 | AWIV2 | Gotjawal | AW | 4.7 | 55.0 | 1.3 | 31.4 | 7.4 | 1.0 | 57.9 | 71.0 | 31.9 | 2.1 | 15.0 |
| 11 | AWIV3 | Gotjawal | AW | 5.3 | 56.5 | 1.8 | 52.1 | 10.5 | 1.4 | 67.5 | 97.3 | 32.7 | 2.2 | 14.6 |
| 12 | AWIV4 | Gotjawal | AW | 4.8 | 45.6 | 0.8 | 28.4 | 5.1 | 0.6 | 47.9 | 72.9 | 26.4 | 1.8 | 14.9 |
| 13 | AWIV5 | Gotjawal | AW | 4.9 | 46.7 | 1.2 | 29.0 | 6.8 | 0.8 | 56.4 | 67.1 | 27.1 | 1.8 | 14.9 |
| 14 | AWIV6 | Gotjawal | AW | 5.1 | 54.4 | 1.8 | 41.0 | 9.5 | 1.1 | 61.3 | 87.2 | 31.5 | 2.3 | 14.0 |
| 15 | GSI_1 | Gotjawal | GS | 4.7 | 37.7 | 0.7 | 15.8 | 4.0 | 0.4 | 52.6 | 39.8 | 21.9 | 1.5 | 14.5 |
| 16 | GSI_2 | Gotjawal | GS | 5.0 | 30.7 | 0.7 | 14.1 | 3.6 | 0.5 | 51.4 | 36.8 | 17.8 | 1.4 | 13.0 |
| 17 | GSI_3 | Gotjawal | GS | 4.8 | 38.6 | 1.0 | 27.0 | 5.9 | 0.5 | 55.0 | 62.6 | 22.4 | 1.8 | 12.8 |
| 18 | GSI_4 | Gotjawal | GS | 5.0 | 35.2 | 0.8 | 16.7 | 4.1 | 0.3 | 46.9 | 46.7 | 20.4 | 1.4 | 14.9 |
| 19 | GSI_5 | Gotjawal | GS | 4.7 | 35.8 | 0.7 | 8.2 | 4.0 | 0.5 | 50.7 | 26.3 | 20.7 | 1.3 | 15.6 |
| 20 | GSI_6 | Gotjawal | GS | 5.1 | 31.2 | 0.4 | 10.3 | 4.6 | 0.4 | 51.4 | 30.5 | 18.1 | 1.1 | 16.6 |
| 21 | GSII_1 | Gotjawal | GS | 5.3 | 28.3 | 0.5 | 10.8 | 4.1 | 0.6 | 47.4 | 33.8 | 16.4 | 1.3 | 12.4 |
| 22 | GSII_2 | Gotjawal | GS | 5.5 | 33.6 | 0.8 | 19.6 | 3.9 | 0.4 | 44.0 | 56.0 | 19.5 | 1.4 | 14.1 |
| 23 | GSII_3 | Gotjawal | GS | 5.4 | 25.2 | 0.4 | 13.1 | 3.6 | 0.5 | 53.9 | 32.7 | 14.6 | 1.2 | 12.6 |
| 24 | GSII_4 | Gotjawal | GS | 5.6 | 25.3 | 0.6 | 11.3 | 4.7 | 0.5 | 54.8 | 31.2 | 14.6 | 1.3 | 11.5 |
| 25 | GSII_5 | Gotjawal | GS | 5.6 | 35.4 | 1.0 | 30.2 | 4.9 | 0.4 | 52.1 | 70.1 | 20.6 | 1.3 | 15.9 |
| 26 | GSII_6 | Gotjawal | GS | 5.1 | 21.7 | 0.5 | 5.4 | 2.9 | 0.4 | 40.5 | 23.0 | 12.6 | 0.9 | 13.5 |
| 27 | GSIV1 | Gotjawal | GS | 5.1 | 41.7 | 1.2 | 22.7 | 4.1 | 0.8 | 51.3 | 56.2 | 24.2 | 1.7 | 14.2 |
| 28 | GSIV2 | Gotjawal | GS | 5.0 | 51.4 | 1.0 | 27.9 | 5.6 | 1.0 | 48.9 | 72.7 | 29.8 | 2.1 | 14.4 |
| 29 | GSIV3 | Gotjawal | GS | 5.1 | 34.7 | 0.7 | 18.4 | 3.5 | 0.8 | 55.9 | 41.9 | 20.1 | 1.4 | 13.9 |
| 30 | GSIV4 | Gotjawal | GS | 5.4 | 32.9 | 0.7 | 24.9 | 5.0 | 0.7 | 53.0 | 59.1 | 19.1 | 1.5 | 13.0 |
| 31 | GSIV5 | Gotjawal | GS | 5.6 | 28.5 | 0.8 | 24.1 | 5.5 | 0.7 | 53.2 | 58.5 | 16.5 | 1.2 | 13.4 |
| 32 | GSIV6 | Gotjawal | GS | 5.6 | 26.6 | 0.7 | 15.2 | 5.0 | 0.7 | 53.0 | 40.7 | 15.4 | 1.3 | 12.2 |
| 33 | KRI_1 | Gotjawal | KR | 5.0 | 12.0 | 0.4 | 3.8 | 2.4 | 0.5 | 49.6 | 14.1 | 7.0 | 0.9 | 8.0 |
| 34 | KRI_2 | Gotjawal | KR | 4.6 | 26.9 | 0.5 | 2.0 | 4.8 | 0.4 | 56.5 | 13.7 | 15.6 | 1.2 | 13.5 |
| 35 | KRI_3 | Gotjawal | KR | 4.9 | 19.7 | 0.4 | 13.9 | 2.7 | 0.3 | 52.1 | 33.3 | 11.5 | 1.0 | 11.6 |
| 36 | KRI_4 | Gotjawal | KR | 4.2 | 23.1 | 0.6 | 12.4 | 3.5 | 0.6 | 49.7 | 34.5 | 13.4 | 0.9 | 14.8 |
| 37 | KRI_5 | Gotjawal | KR | 4.5 | 15.0 | 0.3 | 15.6 | 4.7 | 0.4 | 40.1 | 52.4 | 8.7 | 1.0 | 8.6 |
| 38 | KRI_6 | Gotjawal | KR | 4.7 | 22.1 | 0.5 | 13.0 | 4.0 | 0.4 | 38.1 | 47.0 | 12.8 | 1.0 | 13.1 |
| 39 | KRII_1 | Gotjawal | KR | 4.2 | 29.1 | 0.8 | 11.5 | 2.8 | 0.4 | 29.5 | 52.4 | 16.9 | 1.6 | 10.2 |
| 40 | KRII_2 | Gotjawal | KR | 4.7 | 15.8 | 0.4 | 10.1 | 1.8 | 0.3 | 28.1 | 44.8 | 9.2 | 1.1 | 8.2 |
| 41 | KRII_3 | Gotjawal | KR | 4.2 | 16.3 | 0.2 | 2.4 | 0.6 | 0.2 | 36.6 | 9.3 | 9.4 | 1.0 | 9.3 |
| 42 | KRII_4 | Gotjawal | KR | 4.4 | 25.3 | 0.5 | 13.2 | 3.3 | 0.4 | 28.8 | 60.3 | 14.7 | 1.2 | 11.7 |
| 43 | KRII_5 | Gotjawal | KR | 4.1 | 21.0 | 0.4 | 8.7 | 2.9 | 0.3 | 32.0 | 38.4 | 12.2 | 1.1 | 11.6 |
| 44 | KRII_6 | Gotjawal | KR | 4.4 | 20.7 | 0.4 | 12.6 | 3.6 | 0.4 | 29.4 | 57.5 | 12.0 | 1.1 | 10.5 |
| 45 | KRIII_1 | Gotjawal | KR | 5.2 | 28.2 | 1.0 | 22.4 | 0.0 | 0.3 | 39.9 | 59.4 | 16.3 | 1.2 | 13.5 |
| 46 | KRIII_2 | Gotjawal | KR | 5.7 | 24.7 | 0.8 | 28.1 | 0.5 | 0.2 | 44.4 | 66.6 | 14.3 | 1.2 | 11.8 |
| 47 | KRIII_3 | Gotjawal | KR | 5.2 | 21.5 | 0.6 | 14.3 | 0.3 | 0.2 | 46.2 | 33.2 | 12.4 | 1.1 | 11.2 |
| 48 | KRIII_4 | Gotjawal | KR | 5.4 | 22.7 | 0.7 | 19.5 | 7.2 | 0.3 | 43.1 | 64.1 | 13.2 | 1.1 | 12.0 |
| 49 | KRIII_5 | Gotjawal | KR | 5.1 | 38.3 | 0.7 | 24.7 | 0.1 | 0.2 | 37.4 | 68.7 | 22.2 | 1.0 | 21.8 |
| 50 | KRIII_6 | Gotjawal | KR | 5.3 | 28.6 | 0.7 | 28.9 | 0.1 | 0.3 | 33.2 | 90.4 | 16.6 | 1.0 | 16.7 |
| 51 | KRIV_1 | Gotjawal | KR | 4.5 | 22.7 | 0.7 | 11.3 | 2.5 | 0.3 | 40.5 | 36.6 | 13.2 | 1.1 | 11.8 |
| 52 | KRIV_2 | Gotjawal | KR | 4.7 | 23.8 | 0.8 | 20.1 | 3.9 | 0.5 | 48.7 | 52.1 | 13.8 | 1.4 | 10.2 |
| 53 | KRIV_3 | Gotjawal | KR | 4.7 | 22.8 | 0.7 | 9.7 | 2.3 | 0.4 | 49.8 | 26.4 | 13.2 | 1.3 | 10.0 |
| 54 | KRIV_4 | Gotjawal | KR | 4.6 | 23.1 | 0.9 | 19.8 | 5.1 | 0.6 | 50.9 | 51.7 | 13.4 | 1.1 | 11.7 |
| 55 | KRIV_5 | Gotjawal | KR | 4.3 | 24.3 | 0.8 | 19.2 | 4.3 | 0.6 | 40.2 | 61.8 | 14.1 | 1.0 | 13.9 |
| 56 | KRIV_6 | Gotjawal | KR | 4.3 | 18.9 | 0.5 | 12.0 | 2.9 | 0.3 | 32.1 | 49.2 | 10.9 | 0.8 | 12.9 |
| 57 | SYI_1 | Gotjawal | SY | 4.7 | 25.3 | 0.6 | 25.8 | 7.4 | 0.4 | 55.7 | 61.3 | 14.7 | 1.0 | 14.5 |
| 58 | SYI_2 | Gotjawal | SY | 5.1 | 21.7 | 0.6 | 33.3 | 6.8 | 0.4 | 45.6 | 90.1 | 12.6 | 1.1 | 11.6 |
| 59 | SYI_3 | Gotjawal | SY | 5.0 | 23.6 | 0.6 | 28.1 | 6.8 | 0.5 | 49.9 | 72.0 | 13.7 | 1.0 | 14.3 |
| 60 | SYI_4 | Gotjawal | SY | 5.0 | 21.5 | 0.6 | 21.3 | 7.1 | 0.6 | 65.3 | 45.4 | 12.5 | 1.1 | 11.7 |
| 61 | SYI_6 | Gotjawal | SY | 5.2 | 19.5 | 0.6 | 16.3 | 4.6 | 0.4 | 54.5 | 40.3 | 11.3 | 1.4 | 7.9 |
| 62 | SYII_1 | Gotjawal | SY | 5.1 | 26.0 | 0.6 | 13.7 | 6.6 | 0.4 | 20.2 | 105.4 | 15.1 | 1.3 | 11.3 |
| 63 | SYII_2 | Gotjawal | SY | 5.0 | 24.1 | 0.5 | 11.8 | 3.9 | 0.4 | 20.8 | 79.7 | 14.0 | 1.2 | 11.3 |
| 64 | SYII_3 | Gotjawal | SY | 5.7 | 28.7 | 0.5 | 29.9 | 4.4 | 0.3 | 45.8 | 76.6 | 16.7 | 1.3 | 13.2 |
| 65 | SYII_4 | Gotjawal | SY | 6.1 | 29.2 | 0.7 | 38.3 | 5.9 | 0.4 | 46.9 | 96.5 | 16.9 | 1.4 | 12.5 |
| 66 | SYII_5 | Gotjawal | SY | 6.0 | 26.8 | 0.7 | 38.5 | 5.9 | 0.4 | 36.0 | 126.4 | 15.6 | 1.4 | 10.8 |
| 67 | SYII_6 | Gotjawal | SY | 5.4 | 25.7 | 0.7 | 20.9 | 5.2 | 0.4 | 24.6 | 110.9 | 14.9 | 1.3 | 11.5 |
| 68 | SYIII_1 | Gotjawal | SY | 5.9 | 22.0 | 0.8 | 21.0 | 0.7 | 0.4 | 39.4 | 58.2 | 12.8 | 1.1 | 12.1 |
| 69 | SYIII_2 | Gotjawal | SY | 5.6 | 31.9 | 0.8 | 34.2 | 0.8 | 0.3 | 36.0 | 100.0 | 18.5 | 1.1 | 17.5 |
| 70 | SYIII_3 | Gotjawal | SY | 6.5 | 23.7 | 0.9 | 48.9 | 0.1 | 0.3 | 44.2 | 113.6 | 13.7 | 1.0 | 13.5 |
| 71 | SYIII_4 | Gotjawal | SY | 5.1 | 31.2 | 1.0 | 15.6 | 0.6 | 0.3 | 52.8 | 33.0 | 18.1 | 1.3 | 14.4 |
| 72 | SYIII_5 | Gotjawal | SY | 6.3 | 28.2 | 1.1 | 44.3 | 0.1 | 0.3 | 43.0 | 106.4 | 16.3 | 1.1 | 14.3 |
| 73 | SYIII_6 | Gotjawal | SY | 4.8 | 30.9 | 0.8 | 9.3 | 7.2 | 0.8 | 41.2 | 43.9 | 17.9 | 1.4 | 12.9 |
| 74 | SYIV_1 | Gotjawal | SY | 5.7 | 23.3 | 0.7 | 31.1 | 6.9 | 0.5 | 42.2 | 93.0 | 13.5 | 0.8 | 16.4 |
| 75 | SYIV_2 | Gotjawal | SY | 5.4 | 19.3 | 0.6 | 26.7 | 4.3 | 0.4 | 41.5 | 77.2 | 11.2 | 0.8 | 13.2 |
| 76 | SYIV_3 | Gotjawal | SY | 4.8 | 20.7 | 0.6 | 13.0 | 3.5 | 0.4 | 36.8 | 47.4 | 12.0 | 0.8 | 14.2 |
| 77 | SYIV_4 | Gotjawal | SY | 4.9 | 25.6 | 0.7 | 21.0 | 4.8 | 0.5 | 46.3 | 58.4 | 14.8 | 1.4 | 10.8 |
| 78 | SYIV_5 | Gotjawal | SY | 5.1 | 22.7 | 0.5 | 12.7 | 5.4 | 0.7 | 42.9 | 45.0 | 13.2 | 1.1 | 11.6 |
| 79 | SYIV_6 | Gotjawal | SY | 4.9 | 32.0 | 1.1 | 25.2 | 5.6 | 0.7 | 44.5 | 72.9 | 18.6 | 1.4 | 13.0 |
| 80 | C2I_1 | Jeju | HL2 | 4.4 | 14.7 | 0.2 | 0.9 | 0.3 | 0.2 | 32.3 | 5.2 | 8.6 | 0.5 | 17.2 |
| 81 | C2I_2 | Jeju | HL2 | 4.2 | 12.9 | 0.2 | 0.9 | 0.2 | 0.2 | 37.2 | 4.1 | 7.5 | 0.5 | 16.5 |
| 82 | C2I_3 | Jeju | HL2 | 4.5 | 12.5 | 0.2 | 1.6 | 0.5 | 0.3 | 34.9 | 7.5 | 7.3 | 0.4 | 17.1 |
| 83 | C2I_4 | Jeju | HL2 | 4.3 | 16.0 | 0.3 | 1.4 | 0.5 | 0.2 | 35.5 | 6.9 | 9.3 | 0.5 | 18.5 |
| 84 | C2I_5 | Jeju | HL2 | 4.1 | 16.0 | 0.2 | 0.5 | 0.2 | 0.2 | 42.3 | 2.5 | 9.3 | 0.4 | 20.8 |
| 85 | C2I_6 | Jeju | HL2 | 4.3 | 12.6 | 0.3 | 1.0 | 0.4 | 0.3 | 40.3 | 5.0 | 7.3 | 0.5 | 14.3 |
| 86 | C2II_1 | Jeju | HL2 | 4.2 | 14.5 | 0.1 | 1.0 | 0.3 | 0.1 | 24.0 | 6.1 | 8.4 | 0.6 | 13.8 |
| 87 | C2II_2 | Jeju | HL2 | 4.2 | 14.6 | 0.1 | 0.8 | 0.2 | 0.1 | 21.0 | 6.0 | 8.5 | 0.6 | 13.9 |
| 88 | C2II_3 | Jeju | HL2 | 4.1 | 12.5 | 0.1 | 0.4 | 0.2 | 0.1 | 20.0 | 4.1 | 7.2 | 0.6 | 12.3 |
| 89 | C2II_4 | Jeju | HL2 | 4.1 | 15.2 | 0.1 | 0.7 | 0.3 | 0.1 | 23.8 | 5.2 | 8.8 | 0.6 | 15.2 |
| 90 | C2II_5 | Jeju | HL2 | 4.2 | 13.3 | 0.1 | 1.1 | 0.3 | 0.1 | 23.1 | 7.2 | 7.7 | 0.5 | 14.2 |
| 91 | C2II_6 | Jeju | HL2 | 4.1 | 15.0 | 0.1 | 0.8 | 0.3 | 0.1 | 20.7 | 6.2 | 8.7 | 0.4 | 22.6 |
| 92 | C2IV1 | Jeju | HL2 | 4.1 | 16.1 | 0.2 | 1.9 | 0.5 | 0.2 | 28.4 | 9.9 | 9.3 | 0.8 | 11.4 |
| 93 | C2IV2 | Jeju | HL2 | 4.0 | 16.4 | 0.2 | 0.8 | 0.3 | 0.2 | 26.1 | 5.4 | 9.5 | 0.8 | 11.9 |
| 94 | C2IV3 | Jeju | HL2 | 4.1 | 14.4 | 0.2 | 1.2 | 0.4 | 0.2 | 30.2 | 6.4 | 8.3 | 0.8 | 10.4 |
| 95 | C2IV4 | Jeju | HL2 | 4.1 | 14.9 | 0.3 | 2.2 | 0.6 | 0.2 | 27.7 | 11.9 | 8.7 | 0.8 | 10.4 |
| 96 | C2IV5 | Jeju | HL2 | 4.1 | 13.3 | 0.2 | 1.3 | 0.5 | 0.2 | 29.0 | 7.5 | 7.7 | 0.7 | 10.7 |
| 97 | C2IV6 | Jeju | HL2 | 4.0 | 13.7 | 0.2 | 0.7 | 0.3 | 0.2 | 46.3 | 2.9 | 7.9 | 0.8 | 10.4 |
| 98 | CI_1 | Jeju | HL1 | 4.5 | 31.2 | 0.9 | 6.2 | 2.6 | 0.2 | 44.1 | 22.3 | 18.1 | 1.2 | 15.6 |
| 99 | CI_2 | Jeju | HL1 | 4.6 | 26.5 | 0.5 | 1.5 | 0.9 | 0.2 | 38.2 | 8.0 | 15.4 | 0.9 | 17.2 |
| 100 | CI_3 | Jeju | HL1 | 4.4 | 24.6 | 0.7 | 1.7 | 0.9 | 0.2 | 38.8 | 8.8 | 14.3 | 0.9 | 15.6 |
| 101 | CI_4 | Jeju | HL1 | 4.6 | 26.6 | 0.8 | 7.2 | 3.5 | 0.2 | 39.3 | 30.0 | 15.4 | 1.1 | 14.7 |
| 102 | CI_5 | Jeju | HL1 | 4.5 | 23.9 | 0.7 | 4.1 | 2.0 | 0.2 | 37.2 | 18.7 | 13.9 | 0.9 | 14.9 |
| 103 | CI_6 | Jeju | HL1 | 4.6 | 27.8 | 0.6 | 5.4 | 2.7 | 0.2 | 41.4 | 21.6 | 16.1 | 0.9 | 17.8 |
| 104 | CII_1 | Jeju | HL1 | 5.0 | 17.5 | 0.2 | 2.1 | 1.1 | 2.2 | 31.6 | 17.7 | 10.2 | 0.7 | 15.2 |
| 105 | CII_2 | Jeju | HL1 | 5.0 | 20.2 | 0.5 | 7.8 | 2.9 | 0.3 | 39.6 | 28.9 | 11.7 | 0.7 | 17.9 |
| 106 | CII_3 | Jeju | HL1 | 4.5 | 25.1 | 0.2 | 2.1 | 1.3 | 0.2 | 36.2 | 10.5 | 14.6 | 1.0 | 15.1 |
| 107 | CII_4 | Jeju | HL1 | 4.6 | 23.8 | 0.2 | 1.4 | 0.6 | 0.2 | 42.6 | 5.8 | 13.8 | 0.7 | 19.5 |
| 108 | CII_5 | Jeju | HL1 | 4.8 | 18.2 | 0.2 | 0.8 | 0.4 | 0.2 | 37.3 | 4.5 | 10.6 | 0.6 | 18.8 |
| 109 | CII_6 | Jeju | HL1 | 4.6 | 25.0 | 0.5 | 4.7 | 1.9 | 0.2 | 30.3 | 24.1 | 14.5 | 0.8 | 19.2 |
| 110 | CIV_2 | Jeju | HL1 | 5.0 | 22.3 | 0.6 | 6.2 | 1.8 | 0.4 | 45.8 | 19.7 | 12.9 | 0.9 | 13.7 |
| 111 | CIV_3 | Jeju | HL1 | 4.8 | 27.1 | 0.4 | 1.6 | 0.8 | 0.3 | 47.0 | 6.7 | 15.7 | 1.0 | 15.8 |
| 112 | CIV_4 | Jeju | HL1 | 4.6 | 22.8 | 0.6 | 5.9 | 1.4 | 0.4 | 47.9 | 17.1 | 13.2 | 1.2 | 11.4 |
| 113 | CIV_5 | Jeju | HL1 | 5.1 | 16.3 | 1.0 | 11.6 | 3.3 | 0.5 | 45.7 | 35.8 | 9.5 | 1.1 | 8.4 |
| 114 | CIV_6 | Jeju | HL1 | 5.1 | 22.2 | 0.4 | 3.7 | 1.2 | 0.3 | 37.2 | 15.1 | 12.9 | 0.5 | 24.9 |

Supplementary Table 3. The number of reads that passed quality controls are summarized.

| Sample | Code | ID | Raw reads | Pandas | Sequence Filter | FrameBot |
| --- | --- | --- | --- | --- | --- | --- |
| AWI-1 | Gotjawal | AW | 199,628 | 189,759 | 187,723 | 182,404 |
| AWI-3 | Gotjawal | AW | 105,087 | 91,729 | 90,867 | 84,354 |
| AWI-4 | Gotjawal | AW | 125,474 | 111,919 | 110,254 | 102,999 |
| AWI-5 | Gotjawal | AW | 80,023 | 63,866 | 62,212 | 51,664 |
| AWI-6 | Gotjawal | AW | 80,345 | 65,995 | 64,182 | 52,558 |
| AWII-1 | Gotjawal | AW | 195,099 | 180,344 | 179,450 | 165,950 |
| AWII-3 | Gotjawal | AW | 110,907 | 103,975 | 103,671 | 100,055 |
| AWII-5 | Gotjawal | AW | 179,724 | 164,641 | 164,069 | 151,390 |
| AWIV1 | Gotjawal | AW | 70,910 | 61,699 | 58,354 | 46,579 |
| AWIV2 | Gotjawal | AW | 43,314 | 33,078 | 29,998 | 23,171 |
| AWIV3 | Gotjawal | AW | 60,405 | 49,135 | 45,213 | 36,504 |
| AWIV4 | Gotjawal | AW | 59,720 | 47,274 | 44,389 | 37,463 |
| AWIV5 | Gotjawal | AW | 72,142 | 62,989 | 59,578 | 49,869 |
| AWIV6 | Gotjawal | AW | 53,768 | 44,494 | 39,822 | 30,006 |
| C2I-1 | Jeju | HL1 | 170,933 | 159,179 | 152,784 | 146,011 |
| C2I-2 | Jeju | HL1 | 148,629 | 136,026 | 134,914 | 129,014 |
| C2I-3 | Jeju | HL1 | 223,060 | 208,606 | 207,267 | 201,526 |
| C2I-4 | Jeju | HL1 | 143,613 | 127,893 | 126,326 | 119,867 |
| C2I-5 | Jeju | HL1 | 152,935 | 138,512 | 136,992 | 130,742 |
| C2I-6 | Jeju | HL1 | 192,543 | 178,882 | 177,868 | 170,605 |
| C2II-1 | Jeju | HL1 | 235,216 | 217,685 | 216,397 | 207,642 |
| C2II-2 | Jeju | HL1 | 130,466 | 121,640 | 121,242 | 120,087 |
| C2II-3 | Jeju | HL1 | 254,872 | 238,409 | 237,611 | 230,743 |
| C2II-4 | Jeju | HL1 | 299,609 | 280,908 | 279,690 | 272,900 |
| C2II-5 | Jeju | HL1 | 309,136 | 291,604 | 290,414 | 286,775 |
| C2II-6 | Jeju | HL1 | 285,422 | 270,912 | 270,065 | 266,175 |
| C2IV2 | Jeju | HL1 | 30,686 | 26,945 | 25,338 | 17,837 |
| C2IV3 | Jeju | HL1 | 135,196 | 122,682 | 119,822 | 112,960 |
| C2IV4 | Jeju | HL1 | 130,682 | 117,903 | 115,142 | 108,132 |
| C2IV5 | Jeju | HL1 | 129,491 | 118,456 | 115,399 | 108,555 |
| C2IV6 | Jeju | HL1 | 78,895 | 63,441 | 61,253 | 54,440 |
| CI-1 | Jeju | HL2 | 65,344 | 20,967 | 17,810 | 14,086 |
| CI-2 | Jeju | HL2 | 67,182 | 23,600 | 20,869 | 15,338 |
| CI-3 | Jeju | HL2 | 72,433 | 41,185 | 40,243 | 35,574 |
| CI-4 | Jeju | HL2 | 55,340 | 20,797 | 18,200 | 14,360 |
| CI-5 | Jeju | HL2 | 63,218 | 24,083 | 21,342 | 17,492 |
| CI-6 | Jeju | HL2 | 73,186 | 35,217 | 33,829 | 27,621 |
| CII-1 | Jeju | HL2 | 57,691 | 17,496 | 14,687 | 10,569 |
| CII-2 | Jeju | HL2 | 57,971 | 20,534 | 17,364 | 13,840 |
| CII-3 | Jeju | HL2 | 83,568 | 33,310 | 29,835 | 23,941 |
| CII-4 | Jeju | HL2 | 97,661 | 69,986 | 69,518 | 65,159 |
| CII-5 | Jeju | HL2 | 65,739 | 30,497 | 29,015 | 23,952 |
| CII-6 | Jeju | HL2 | 102,975 | 82,470 | 82,377 | 78,748 |
| CIV-1 | Jeju | HL2 | 65,191 | 24,078 | 20,484 | 15,731 |
| CIV-2 | Jeju | HL2 | 65,739 | 24,521 | 21,776 | 16,687 |
| CIV-3 | Jeju | HL2 | 66,567 | 22,211 | 17,711 | 13,220 |
| CIV-4 | Jeju | HL2 | 65,014 | 21,207 | 17,075 | 12,531 |
| CIV-5 | Jeju | HL2 | 68,115 | 24,108 | 20,696 | 15,432 |
| CIV-6 | Jeju | HL2 | 70,142 | 27,754 | 25,186 | 19,707 |
| GSI-1 | Gotjawal | GS | 234,077 | 219,686 | 218,379 | 212,907 |
| GSI-2 | Gotjawal | GS | 107,753 | 91,913 | 90,914 | 80,619 |
| GSI-3 | Gotjawal | GS | 93,458 | 80,470 | 79,396 | 70,398 |
| GSI-4 | Gotjawal | GS | 87,516 | 73,911 | 72,481 | 60,631 |
| GSI-5 | Gotjawal | GS | 121,335 | 108,613 | 107,396 | 98,833 |
| GSI-6 | Gotjawal | GS | 91,939 | 76,062 | 74,393 | 63,167 |
| GSII-1 | Gotjawal | GS | 187,970 | 173,189 | 172,133 | 154,453 |
| GSII-2 | Gotjawal | GS | 255,217 | 243,595 | 242,795 | 239,780 |
| GSII-3 | Gotjawal | GS | 203,224 | 187,625 | 186,875 | 166,159 |
| GSII-4 | Gotjawal | GS | 218,616 | 204,240 | 203,482 | 188,754 |
| GSII-5 | Gotjawal | GS | 199,813 | 184,157 | 183,281 | 158,338 |
| GSII-6 | Gotjawal | GS | 170,395 | 157,427 | 156,763 | 142,558 |
| GSIV1 | Gotjawal | GS | 70,890 | 61,192 | 58,799 | 46,369 |
| GSIV2 | Gotjawal | GS | 95,205 | 80,772 | 77,486 | 71,473 |
| GSIV3 | Gotjawal | GS | 69,191 | 60,972 | 54,416 | 42,131 |
| GSIV4 | Gotjawal | GS | 56,004 | 47,726 | 42,073 | 29,374 |
| GSIV5 | Gotjawal | GS | 70,996 | 62,057 | 58,032 | 44,907 |
| GSIV6 | Gotjawal | GS | 103,820 | 92,182 | 82,334 | 37,087 |
| KRI-1 | Gotjawal | KR | 103,376 | 65,452 | 64,704 | 52,448 |
| KRI-2 | Gotjawal | KR | 66,708 | 16,878 | 12,292 | 6,885 |
| KRI-3 | Gotjawal | KR | 57,993 | 12,239 | 9,017 | 3,563 |
| KRI-4 | Gotjawal | KR | 57,428 | 15,024 | 12,569 | 7,230 |
| KRI-5 | Gotjawal | KR | 55,571 | 13,171 | 11,357 | 6,576 |
| KRI-6 | Gotjawal | KR | 64,118 | 18,652 | 15,916 | 10,329 |
| KRII-1 | Gotjawal | KR | 63,681 | 12,593 | 7,096 | 2,395 |
| KRII-2 | Gotjawal | KR | 60,601 | 12,449 | 7,203 | 2,718 |
| KRII-3 | Gotjawal | KR | 72,105 | 16,537 | 11,052 | 5,098 |
| KRII-4 | Gotjawal | KR | 68,978 | 14,449 | 10,696 | 3,574 |
| KRII-5 | Gotjawal | KR | 74,316 | 24,372 | 21,544 | 13,849 |
| KRII-6 | Gotjawal | KR | 56,757 | 12,792 | 8,330 | 4,718 |
| KRIII-1 | Gotjawal | KR | 63,846 | 13,487 | 9,729 | 2,461 |
| KRIII-2 | Gotjawal | KR | 69,198 | 16,854 | 12,732 | 6,678 |
| KRIII-3 | Gotjawal | KR | 69,048 | 14,878 | 9,176 | 2,631 |
| KRIII-4 | Gotjawal | KR | 67,106 | 16,004 | 11,274 | 5,006 |
| KRIII-5 | Gotjawal | KR | 66,614 | 21,459 | 20,283 | 11,922 |
| KRIII-6 | Gotjawal | KR | 55,687 | 11,975 | 8,539 | 2,965 |
| KRIV-1 | Gotjawal | KR | 66,639 | 17,898 | 14,265 | 8,020 |
| KRIV-2 | Gotjawal | KR | 68,211 | 15,732 | 12,518 | 5,296 |
| KRIV-3 | Gotjawal | KR | 58,718 | 12,017 | 7,474 | 2,191 |
| KRIV-4 | Gotjawal | KR | 65,559 | 27,876 | 26,353 | 20,867 |
| KRIV-5 | Gotjawal | KR | 62,301 | 16,338 | 13,345 | 6,862 |
| KRIV-6 | Gotjawal | KR | 62,504 | 19,159 | 17,591 | 10,909 |
| SYI-1 | Gotjawal | SY | 95,124 | 45,382 | 44,495 | 26,577 |
| SYI-2 | Gotjawal | SY | 37,539 | 6,466 | 6,155 | 3,071 |
| SYI-3 | Gotjawal | SY | 71,606 | 31,685 | 30,040 | 24,112 |
| SYI-4 | Gotjawal | SY | 91,853 | 48,342 | 47,212 | 36,515 |
| SYI-6 | Gotjawal | SY | 61,069 | 18,225 | 15,151 | 9,472 |
| SYII-1 | Gotjawal | SY | 68,517 | 15,838 | 9,425 | 4,287 |
| SYII-2 | Gotjawal | SY | 57,265 | 12,669 | 7,222 | 2,798 |
| SYII-3 | Gotjawal | SY | 54,385 | 22,407 | 19,598 | 16,813 |
| SYII-4 | Gotjawal | SY | 77,216 | 20,900 | 14,367 | 8,632 |
| SYII-5 | Gotjawal | SY | 57,097 | 11,357 | 6,200 | 1,278 |
| SYII-6 | Gotjawal | SY | 57,863 | 11,115 | 6,509 | 1,996 |
| SYIII-1 | Gotjawal | SY | 67,283 | 14,329 | 9,380 | 2,494 |
| SYIII-2 | Gotjawal | SY | 65,134 | 13,172 | 10,322 | 1,913 |
| SYIII-3 | Gotjawal | SY | 50,140 | 10,021 | 7,847 | 3,180 |
| SYIII-4 | Gotjawal | SY | 70,754 | 15,229 | 10,611 | 2,621 |
| SYIII-5 | Gotjawal | SY | 54,943 | 11,765 | 7,918 | 2,853 |
| SYIII-6 | Gotjawal | SY | 72,832 | 21,351 | 19,130 | 10,005 |
| SYIV-1 | Gotjawal | SY | 65,699 | 17,623 | 15,108 | 7,071 |
| SYIV-2 | Gotjawal | SY | 73,037 | 23,881 | 22,176 | 12,736 |
| SYIV-3 | Gotjawal | SY | 59,871 | 12,351 | 9,172 | 2,189 |
| SYIV-4 | Gotjawal | SY | 68,293 | 20,330 | 18,720 | 9,988 |
| SYIV-5 | Gotjawal | SY | 55,105 | 12,921 | 9,421 | 3,881 |
| SYIV-6 | Gotjawal | SY | 68,352 | 17,540 | 15,418 | 6,292 |
